# Influence of single-cell RNA sequencing data integration on the performance of differential gene expression analysis

**DOI:** 10.3389/fgene.2022.1009316

**Published:** 2022-11-01

**Authors:** Tomasz Kujawa, Michał Marczyk, Joanna Polanska

**Affiliations:** ^1^ Department of Data Science and Engineering, Silesian University of Technology, Gliwice, Poland; ^2^ Yale Cancer Center, Yale School of Medicine, New Haven, CT, United States

**Keywords:** single-cell RNA sequencing, data integration, batch correction, differential gene expression, joint analysis

## Abstract

Large-scale comprehensive single-cell experiments are often resource-intensive and require the involvement of many laboratories and/or taking measurements at various times. This inevitably leads to batch effects, and systematic variations in the data that might occur due to different technology platforms, reagent lots, or handling personnel. Such technical differences confound biological variations of interest and need to be corrected during the data integration process. Data integration is a challenging task due to the overlapping of biological and technical factors, which makes it difficult to distinguish their individual contribution to the overall observed effect. Moreover, the choice of integration method may impact the downstream analyses, including searching for differentially expressed genes. From the existing data integration methods, we selected only those that return the full expression matrix. We evaluated six methods in terms of their influence on the performance of differential gene expression analysis in two single-cell datasets with the same biological study design that differ only in the way the measurement was done: one dataset manifests strong batch effects due to the measurements of each sample at a different time. Integrated data were visualized using the UMAP method. The evaluation was done both on individual gene level using parametric and non-parametric approaches for finding differentially expressed genes and on gene set level using gene set enrichment analysis. As an evaluation metric, we used two correlation coefficients, Pearson and Spearman, of the obtained test statistics between reference, test, and corrected studies. Visual comparison of UMAP plots highlighted ComBat-seq, limma, and MNN, which reduced batch effects and preserved differences between biological conditions. Most of the tested methods changed the data distribution after integration, which negatively impacts the use of parametric methods for the analysis. Two algorithms, MNN and Scanorama, gave very poor results in terms of differential analysis on gene and gene set levels. Finally, we highlight ComBat-seq as it led to the highest correlation of test statistics between reference and corrected dataset among others. Moreover, it does not distort the original distribution of gene expression data, so it can be used in all types of downstream analyses.

## 1 Introduction

Single-cell RNA sequencing (scRNAseq) is a technique that allows the high-throughput examination of transcriptomes with a single-cell resolution (Lee et al., 2014; Qian et al., 2022). The transcriptome is a dynamic structure that responds rapidly in the form of gene expression to the variety of factors that a cell is subjected to. Moreover, the expression profile can be different in cells of the same type which proves significant cellular heterogeneity (Adil et al., 2021). This heterogeneity is masked in bulk analyses where populations of cells are mixed and sequenced together resulting in signal averages from millions of cells. Single-cell RNA-seq overcomes this barrier and allows the processing of millions of individual cells at a time.

In large projects that involve the processing of many cells data are frequently generated at different times and in different laboratories often equipped with various sequencing platforms (Ming et al., 2022). Combining data generated separately for a consolidated downstream analysis improves statistical power but requires reliable data integration methods. Data integration is also crucial in studies of different omics levels (genomics, proteomics, metabolomics, etc.) to fully understand the molecular complexity of different cell types (Bao et al., 2022). The goal of single-cell data integration is to cluster together cells of similar types; these cells should be intermingled and indistinguishable even if they come from different experiments. In other words, technical differences between datasets should be removed while key biological variations should be preserved. Data integration is a challenging task, especially in large datasets containing highly heterogeneous cell populations. Batch effect removal is a step in which we want to reduce the technical variability in our data that might occur due to differences in sample preparation, sequencing, or processing. Thus, we want to integrate the data that could be assigned to a known batch. Here, we are using the terms data integration and batch correction interchangeably.

There is a variety of distinct algorithms for scRNAseq data integration that are based on different principles and assumptions (Haghverdi et al., 2018; Hie et al., 2019; Lin et al., 2019; Liu et al., 2020; Zhang et al., 2020). An important criterion of the division in terms of our study is based on the output format which can be: (i) full expression matrix; (ii) low-dimensional matrix of embeddings; or (iii) integrated graph. The output type limits the potential downstream applications of integrated data. The full expression matrix is the most versatile format as it could be used in all downstream analyses. On the other hand, a joint embedding is not appropriate for some applications like differential expression analysis or biomarker detection. Hence, the decision about the choice of integration method is crucial and consequential. Another key factor influencing the choice is the main statistical approach that a particular method is based on. We can distinguish two groups here: supervised and unsupervised. The former requires cell-type annotations, and the latter does not rely on data labeling.

The most recent and comprehensive evaluation of scRNAseq integration methods was performed in (Luecken et al., 2022). They evaluated the most popular tools on their ability to remove batch effects while conserving biological information. Their evaluation involved setups with and without cell identity labels and different preprocessing combinations [with/without scaling and highly variable genes (HVGs) selection], as well as the diversity in output formats for each method and task. The conclusion from this work is that there is no single, best integration method and the performance is dependent on the complexity of the integration task (the strength of batch effect, the degree of confounding between batch and biological signals, presence of nuanced biological variation, etc.) (Luecken et al., 2022). Some methods, like BBKNN or Harmony, showed a stronger action towards removing batch effect over conservation of biological variation. For others, like ComBat, MNN, and DESC the trend was in favor of bio-conservation. Deep learning methods that use cell identity information, like scGen or scANVI, preserved biological variation stronger than label-free ones but require larger input data. Generally, HVG selection improved the overall integration performance over the full feature set, except for trajectory and cell-cycle conservation analysis. Scaling the input data typically improved batch removal at a cost of bio-conservation. In another evaluation of scRNAseq data integration methods (Tran et al., 2020), they examined in different simulation scenarios (balanced/unbalanced batches, different dropout rates) the impact of data integration on differential gene expression analysis (DGE analysis), particularly whether it improves the recovery of differentially expressed genes (DEGs). They found that MNN Correct, ZINB-WaVE, ComBat, and scMerge were the top-performing methods. ComBat turned out to be the best method for this task (being one of the worst overall). scMerge had a good balance between DEGs recovery and overall performance.

The above and other benchmarks typically cover a wide range of evaluation aspects such as removal of batch effects and conservation of biological variation, scalability for large datasets, or computational requirements. Regardless of existing comparisons of data integration methods, there is a lack of studies that comprehensively investigate the impact of data integration on differential gene expression analysis using real data. In terms of DGE analysis most studies on benchmarking methods for data integration focus only on overlaps of differentially expressed genes (Chazarra-Gil et al., 2021) not providing deeper insight into the problem. This study aims to fulfill this gap. Using two datasets, one of which requires data integration to correct the confounded design of the study, six integration methods that provide corrected gene expression matrices were compared. The evaluation was done on individual gene level using two different approaches (parametric and non-parametric) and on gene set level (gene set enrichment analysis).

## 2 Materials and methods

### 2.1 Data

The datasets used in this study come from two related scRNAseq experiments aimed at investigating of the effects of navitoclax treatment on the transcriptome of triple-negative breast cancer cell line to better understand the process of developing drug resistance (Marczyk et al., 2020; Patwardhan et al., 2021). In both experiments, the same cancer cell line (MDA-MB-231) was used as a model organism and two biological replicates were provided (A and B). Cells were exposed to 10 µM navitoclax and harvested at 3 time points: before the treatment (baseline; T1), after treatment (T2), and after recovery from the treatment (T3).

In both cases, immediately after plate harvesting, cells were trypsinized and a single-cell suspension at a concentration of 1,000 cells/µl with viability above 90% was prepared. Chromium Single Cell 3′ Library and Gel Bead Kit V2 (PN-120237), Chromium Single Cell A Chip Kit (PN-120236), and Chromium i7 Multiplex Kit (PN-120262) were used to prepare single-cell libraries following the manufacturer’s instructions. The same sequencer was used—HiSeq 4,000 (Illumina). In the first study (Patwardhan et al., 2021) 6,000 cells per sample were used (two samples were multiplexed on one lane) and 25,000 reads per cell were generated. In other study (Marczyk et al., 2020) 1,500 cells/sample were sequenced in one lane generating 200,000 reads/cell.

**FIGURE 1:**
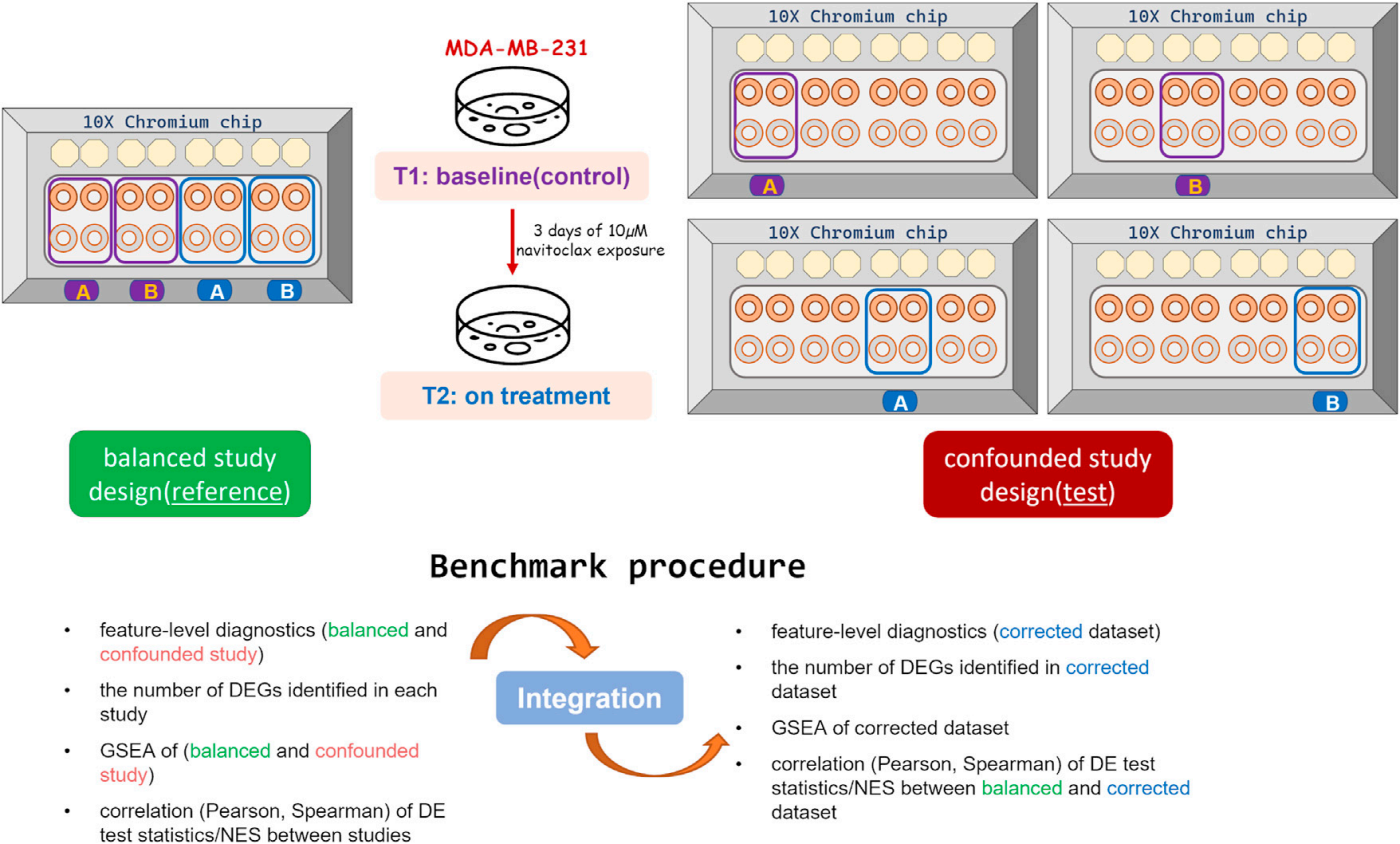
Experimental design and benchmark procedure.

To simplify the evaluation procedure only two time points (T1 and T2) from both datasets (experiments) were considered (Figure 1). Each experiment corresponds to a different design. The first experiment corresponds to a balanced study design where cells collected at different time points were split and processed on the same chip, on the same day (Marczyk et al., 2020). Two biological replicates termed replicate “A” and “B” were involved. This dataset serves as a reference. The second experiment corresponds to a confounded study design where cells collected at different time points were processed on different chips/batches (Patwardhan et al., 2021). This dataset termed a test set was corrected using different data integration methods for the removal of the batch effect.

### 2.2 Data preprocessing

The quality of raw RNA sequencing reads was assessed with FastQC (Andrews, 2010) and the reads were processed with 10x Genomics Cell Ranger 6.1.1 (Zheng et al., 2017) to generate a gene-cell count matrix. Quality control was performed separately for each dataset at cell- and gene-level. Adaptive, sample-specific thresholds were chosen for the number of UMI counts per cell, the number of genes, and the fraction of mitochondrial counts using median absolute deviation (MAD) from the median. Cells were considered of poor quality if a given metric was more than 3 MADs from the median in the wrong direction. Genes that were expressed in less than 1% of cells for each dataset were removed. Finally, we obtained expression matrices with the following dimensions (cells x genes): 12,402 × 4,180 for reference set (Marczyk et al., 2020) and 12,402 × 21,548 for test set (Patwardhan et al., 2021). Such filtered expression matrices were normalized separately using two approaches: deconvolution (Lun et al., 2016) for non-parametric DGE and transcript per million (TPM) metrics for parametric DGE, both followed by (log2+1)-transformation.

Selection of highly variable genes (HVGs) for each dataset was performed using the SCTransform function with variable features. *n* = 5,000 (Hafemeister and Satija, 2019). A common part of 3,620 HVGs was taken as input for data integration. We did not want to be too restrictive with subsampling, as high dimensionality is required for some methods (e.g., to satisfy the orthogonality assumption in MNN detection).

### 2.3 Data integration methods

Since the goal of this study was to evaluate the applicability of scRNAseq data integration methods in terms of further differential analysis, we selected only the methods that: (i) output full corrected expression matrix; (ii) work in an unsupervised manner as we don’t have cell-type labels. Thus, we benchmarked six algorithms (Table 1) and for some of these tested two cases: (i) using all genes; (ii) using only top HVGs.

**TABLE 1:** Selected scRNAseq data integration methods.

Tool	Input	Strategy	Reference
ComBat-seq	raw counts	linear model	Zhang et al. (2020)
limma	logcounts	linear model	Leek et al. (2012)
MNN	logcounts	mutual nearest neighbors (gene expression space)	Haghverdi et al. (2018)
scMerge	logcounts	stably expressed genes + RUV model	Lin et al. (2019)
Seurat	logcounts	canonical correlation analysis + mutual nearest neighbors	Stuart et al. (2019)
Scanorama	raw counts	mutual nearest neighbors (reduced space) + panoraming stiching	Hie et al. (2019)

#### 2.3.1 ComBat-seq

ComBat-seq (Zhang et al., 2020) takes two parameters as input: a raw, untransformed count matrix and a vector describing the annotation of samples into batches. It is also possible to specify biological covariates, whose signals will be preserved in the corrected data. In our case, the technical variable associated with the repetition was used as a batch separation vector and the biological variable was associated with a time point. ComBat-seq uses a negative binomial regression model to estimate batch effects. The computed batch-effect estimators are then used to calculate “batch-freeˮ distributions, i.e., the expected distributions if there were no batch effects in the data based on the model (Zhang et al., 2020). Correction is performed by quantile normalization to make the two distributions (empirical and batch-free) with identical statistical properties. ComBat-seq is the only method that preserves the integer nature of counts making corrected data compatible with various differential expression software (e.g., edgeR, DESeq2).

#### 2.3.2 Limma

Limma (Leek et al., 2012) is another linear method to remove batch effect components from the data. The correction is performed by subtraction of the estimated component from the original data. Limma batch-effect removal function (removeBatchEffect) takes normalized and log-transformed counts as an input. Similarly to ComBat-seq, it allows addition of batch annotations and biological covariates into the model.

#### 2.3.3 Mutual nearest neighbor

MNN searches for mutual nearest neighbors (MNNs) between two datasets or batches in the gene expression space. A pair of MNNs consists of cells present in each batch set of nearest neighbors based on Euclidean distance. These cells are considered to be of the same type/state across batches (Haghverdi et al., 2018). Differences in expression between identified MNNs are used to compute the batch correction vector which is applied to all cells. mnnCorrect function was run with two setups: with all genes and with HVGs. In both cases normalized and log-transformed expression values were used. merge. order, argument was specified such as both repetitions from a given time point were merged first and then combined. Thus, the merging order was as follows: first T1A + T1B and T2A + T2B. Then the summation results were added together. cos.norm. out, was set to FALSE to disable cosine normalization before computing corrected expression values to obtain corrected values on the log scale, similar to the input data. The rest parameters were set to default values.

#### 2.3.4 scMerge

ScMerge (Lin et al., 2019) was run in the unsupervised mode as we do not have cell-type information. In this mode, the estimation of batch effects is performed on two levels: (i) identification of stably expressed genes (SEGs) across batches which serve as “negative control genes”; (ii) k-means clustering based on the HVGs followed by the identification of mutual nearest clusters (MNCs) from the batches based on Pearson correlation as the dissimilarity metric. Cells belonging to a pair of MNCs are considered to be of the same type in different batches and serve as pseudo replicates. SEGs and pseudo replicate information are the inputs for scMerge which uses the RUV model to adjust the data. We ran scMerge with three setups of kmeansK parameter: (5, 5, 5, 5), (4, 4, 4, 4) and (4, 4, 3, 3) on (log2+1)-transformed counts.

#### 2.3.5 Seurat v4

Seurat v4 (Stuart et al., 2019) is another method based on the MNN concept (referred there as “anchors”). This method includes two approaches to match anchors across datasets/batches: Canonical Correlation Analysis (CCA) and reciprocal Principal Component Analysis (rPCA). In both cases, the searching of anchors is performed in a shared, reduced subspace obtained by CCA (linear combinations of genes with the maximum correlation between batches) or rPCA (maximum variation between batches). The correction vector is computed similarly to MNN (difference in expression profiles between two cells in each anchor). The batch integration order is derived from hierarchical clustering based on the distance between the datasets. Seurat v4 (version 4.0) was run according to the data integration tutorial on the web (https://satijalab.org/seurat/articles/integration_introduction.html).

#### 2.3.6 Scanorama

In Scanorama (Hie et al., 2019) the nearest neighbor searching is performed in the low-dimensional subspace obtained by randomized singular value decomposition (SVD). The searching is performed across all batches and the priority of dataset merging is determined based on the percentage of matching cells in the batch. This reduces the risk of overcorrection. Scanorama was run using the reticulate R package following the tutorial (https://github.com/brianhie/scanorama). Two setups were evaluated: with all genes as input and using the top 2,000 HVGs based on data dispersion (internally selected by the algorithm).

### 2.4 Evaluation of data integration methods

#### 2.4.1 Visual inspection of data

UMAP (McInnes and Healy, 2018) was employed for all data visualizations before and after data integration as it performs well at preserving global data structure. UMAP was run with default parameters using runUMAP function from scatter R package (McCarthy et al., 2017).

#### 2.4.2 Differential gene expression analysis: Parametric and non-parametric approaches

Both datasets were processed through the same protocol to find differentially expressed genes using two approaches: the parametric method called MAST (Finak et al., 2015) and the non-parametric method called EMDomics (Nabavi et al., 2016). MAST uses a hurdle model to address bimodal expression distributions in scRNAseq data. The bimodality is manifested in such a way that observed expression is either strongly positive (continuous part) or non-detectable (discrete part). The Hurdle model parameterizes both parts and combines the information from them in the form of gene statistics to infer changes in expression levels. DE testing is performed across the two conditions through the LRT statistic. MAST was applied on the log2 (TPM + 1) expression matrix without including the cellular detection rate (the fraction of genes that are detected with non-zero counts) as a covariate in the model. The following thresholds were used for DEGs identification: an absolute value of log-fold change (LFC) higher than 2, and false discovery rate (FDR) lower than 0.001 (Benjamini–Hochberg method for multiple testing correction was used).

As an alternative when the corrected data do not fit the MAST model, the EMDomics method was used which does not make any assumptions about the data distribution. EMDomics uses the Earth Mover’s distance (EMD) to measure the overall difference between the two normalized distributions (gene expression in two conditions/groups). This method is not restricted to finding only differences in mean expression between two conditions but also captures the overall difference in shape (bimodal vs. unimodal expression) between two distributions. EMDomics was applied to log-normalized counts with default parameters. DEGs were identified based on the following thresholds: emd score higher than 2 and FDR smaller than 0.001. In both cases, cells from two replicates (A and B) were compared between two time points (T1 vs. T2).

A receiver operating characteristic (ROC) curve was created by setting different thresholds on *p*-values from statistical tests while estimating DEGs. To calculate performance metrics, a reference dataset (with a balanced study design) was used as a “ground truth”, and the sensitivity and specificity of each batch correction method were calculated.

#### 2.4.3 GSEA

Differential expression was also performed at the level of gene sets using gene set enrichment analysis (GSEA). This step was done using the fGSEA R package (Korotkevich et al., 2019). DE test statistics obtained by MAST (continuous Z-score: C-component) were used as the ranking metrics. The following gene sets from Molecular Signatures Database (MSigDB) (Liberzon et al., 2015) were tested: Hallmark, Kegg, GO, and REACTOME. The total number of considered gene sets was 12,253; 50 gene sets for Hallmark, 186 for KEGG, 10,402 gene sets for GO, and 1,615 for REACTOME. Gene set was identified as differentially enriched based on a *p*-value lower than 0.05.

#### 2.4.4 Correlation analysis

The correlation analysis was performed both at the level of individual genes (DGE) and gene sets (GSEA). For each data integration method, the previously mentioned DE test statistics were taken: (i) MAST: continuous Z-score (C-component) (ii) EMDomics: emd score (iii) GSEA: normalized enrichment score (NES). The correlation between the balanced (reference) study and the confounded dataset (test set, before correction) was assessed and used as the benchmark for assessing the quality of the data integration (Figure 1). Both, Pearson and Spearman correlation coefficients were calculated.

## 3 Results

### 3.1 Comparison of datasets before data integration

**FIGURE 2:**
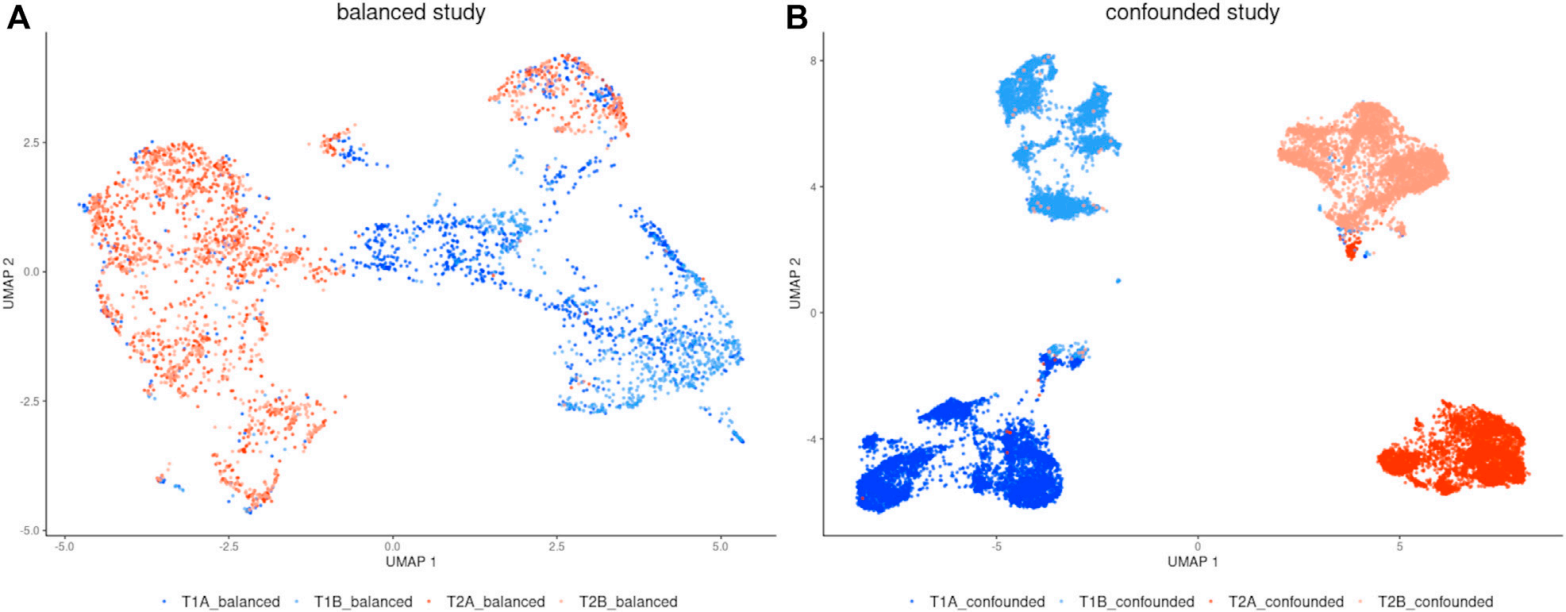
UMAP visualization of **(A)** balanced study, **(B)** confounded study (before correction). There are strong batch effects manifested in the confounded study.

To visually examine the batch effect problem, the UMAP algorithm was run separately for each dataset (Figure 2). In a balanced study design (Figure 2A) there is strong segregation of cells along time points while cells from both repetitions are intermingled, which is desired. The opposite situation is observed in the confounded study design (Figure 2B) where together with separation along time points, the cells group by replicates which proves a strong batch effect. The main cause was that the samples in the confounded study were measured on different days.

Next, we calculated the following properties of individual genes at the single-cell level: mean expression, the variance of expression, and detection rate, which is a proportion of expressed cells (Supplementary Figure S1). We observe a typical situation that could be found in scRNAseq data: up to a mean normalized count of around 1, variance and mean are roughly equal as expected under a Poisson model either for balanced or confounded (before correction) study design. Genes with a higher average expression show overdispersion compared to Poisson distribution (Supplementary Figure S1B,C). As expected in scRNAseq data, in both experiments, many genes are expressed in very few cells. All feature-level statistics were comparable between balanced and confounded studies.

### 3.2 Differential analysis before data integration

The number of DEGs identified with the parametric approach was 965 for balanced and 191 for confounded study. The overlap between the two datasets was 63 genes, from which 43 genes were upregulated, and 20 genes were downregulated in the balanced study, and for the confounded study, the ratio of upregulated to downregulated genes was equal to 20/43. The correlation coefficients were equal to 0.16 (Pearson) and -0.21 (Spearman) and both were significant. After using a non-parametric approach, the number of DEGs was smaller: 80 for balanced and 114 for confounded study. There were no common DEGs between datasets. The correlation between test statistics from both studies was much higher (Spearman: 0.72, Pearson:0.75) than when the parametric method was used.

### 3.3 Data integration for batch effect correction

**FIGURE 3:**
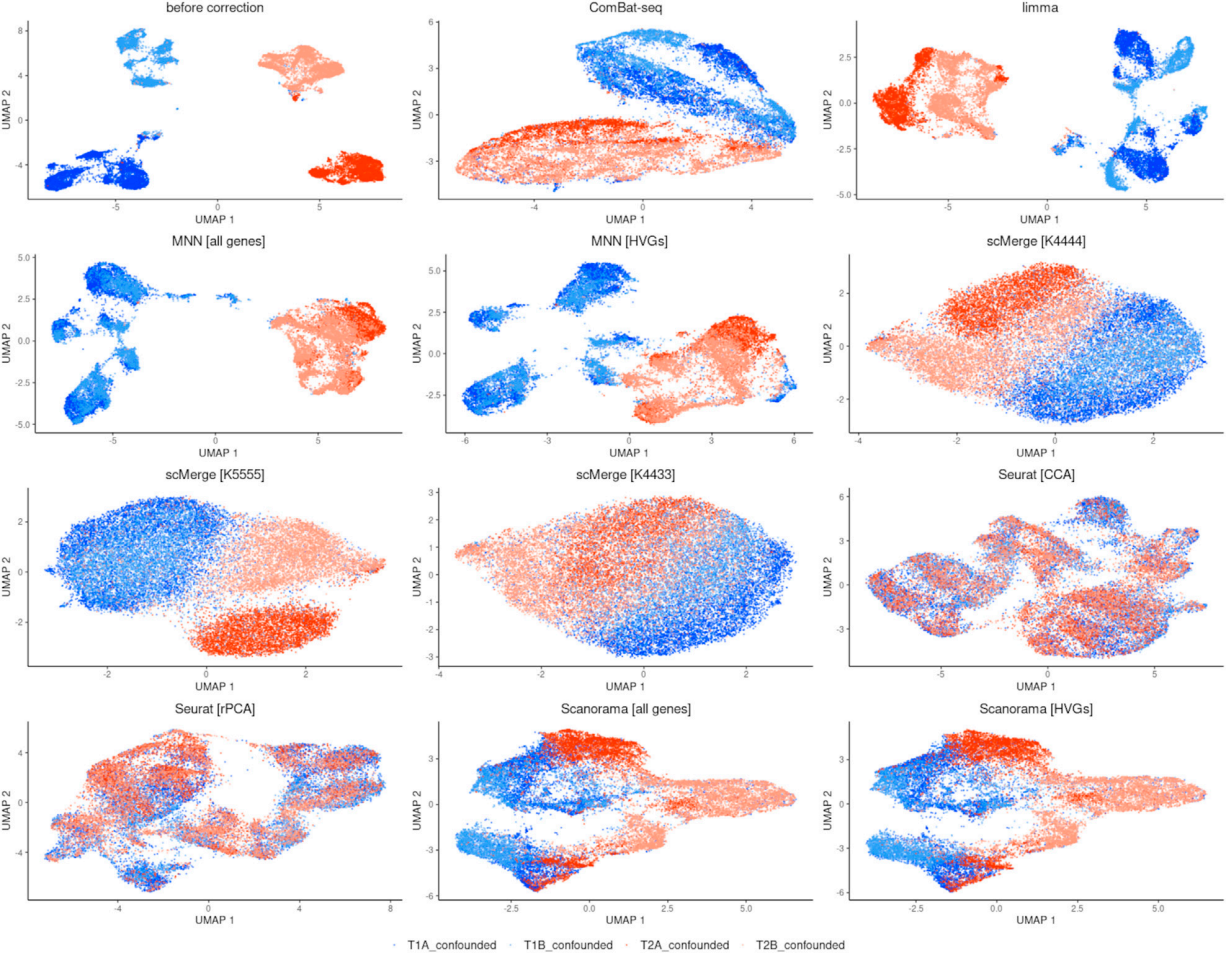
UMAP visualization after integration.

The UMAP plots (Figure 3) show that ComBat-seq might perform best in removing batch effects and preserving biological variation. It produced two strong clusters separated by time point, while the cells from technical repetitions are mixed well. In the case of the limma method, we observe separation by time point, but the repetitions are not mixed well—they seem to have a small tendency to group separately. MNN algorithm improved the separation by time point in both cases when all genes and only the top 3,620 HVGs were taken. However, within the time point T1 cells form characteristic subgroups are observed. scMerge performed visually best with *kmeansK* = (4,4,3,3). In other setups, there is an improvement in separation by time point over no correction, and technical replicates from T1 are well intermingled but not from T2 (replicate A clusters separately from replicate B). Seurat achieved the worst result by mixing all cells together, thus it was not evaluated in further comparisons. Scanorama achieved little improvement no matter if all genes were used or HVGs only.

**FIGURE 4:**
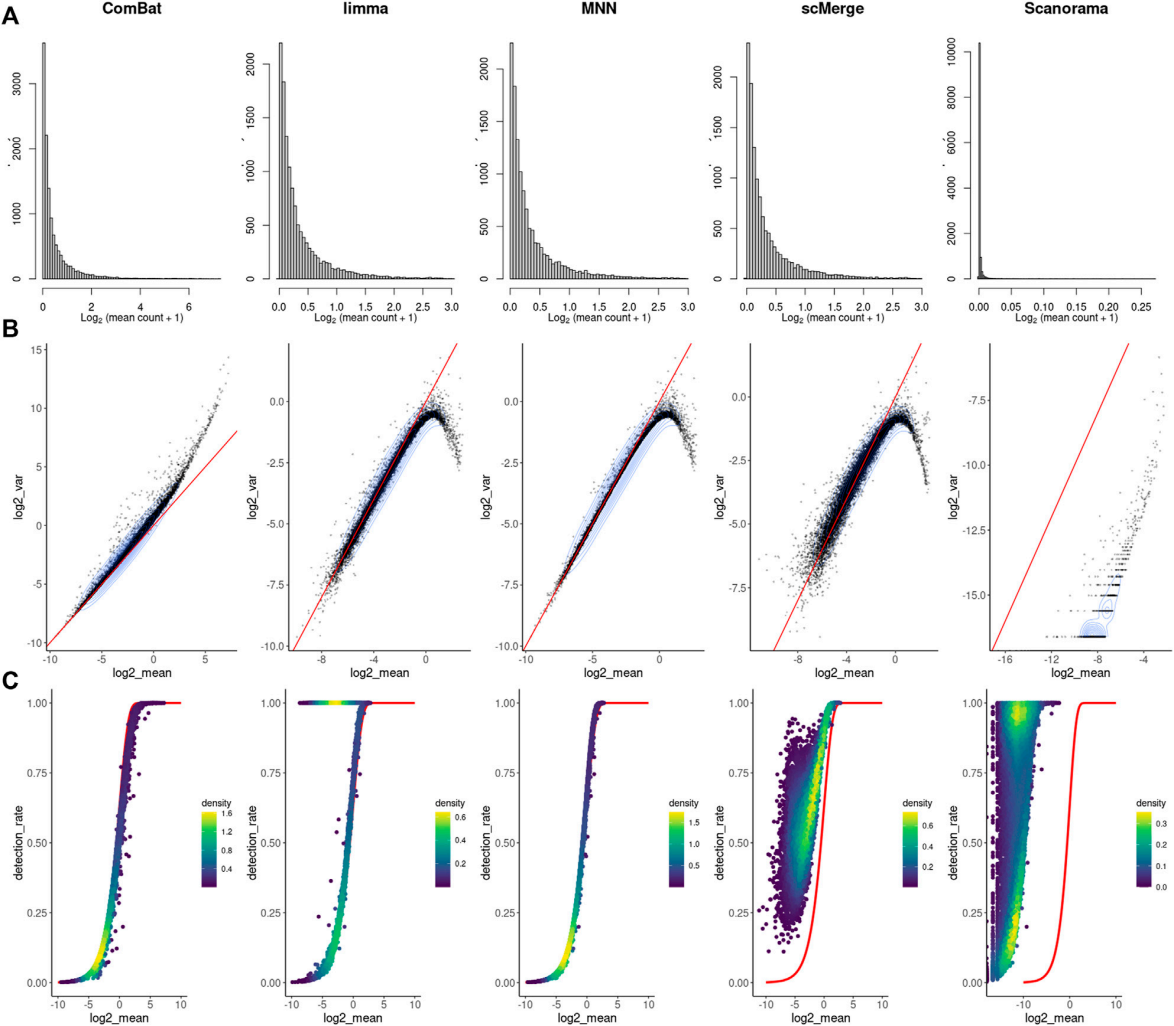
Feature-level metrics for corrected study. **(A)** histograms of mean value, **(B)** mean-variance relationship - red line with intercept = 0 and slope = 1, **(C)** mean-detection rate relationship - red line indicates the expected distribution under a Poisson model. Individual points are colored by the number of neighboring points.

As before, we counted the feature-level metrics after data integration (Figure 4). Except for ComBat-seq, genes with a higher average expression were not following the raw data distribution after correction (Figure 4A). Moreover, for MNN, Scanorama negative values started to occur in the corrected matrix. In most cases, the batch effect correction also distorts the characteristic of the scRNAseq data mean-variance relationship (Figure 4B). There is a sharp collapse of the log variance in the upper range of the mean expression (Figure 4B). The association between average expression and detection rate is conserved only for ComBat-seq and MNN (Figure 4C). Limma introduces small expression values to all cells for many low expression genes (dropout rate equal 1), while scMerge and Scanorama consequently increase dropout rate with increased expression of the gene.

### 3.4 Differential gene expression analysis after data integration

**FIGURE 5:**
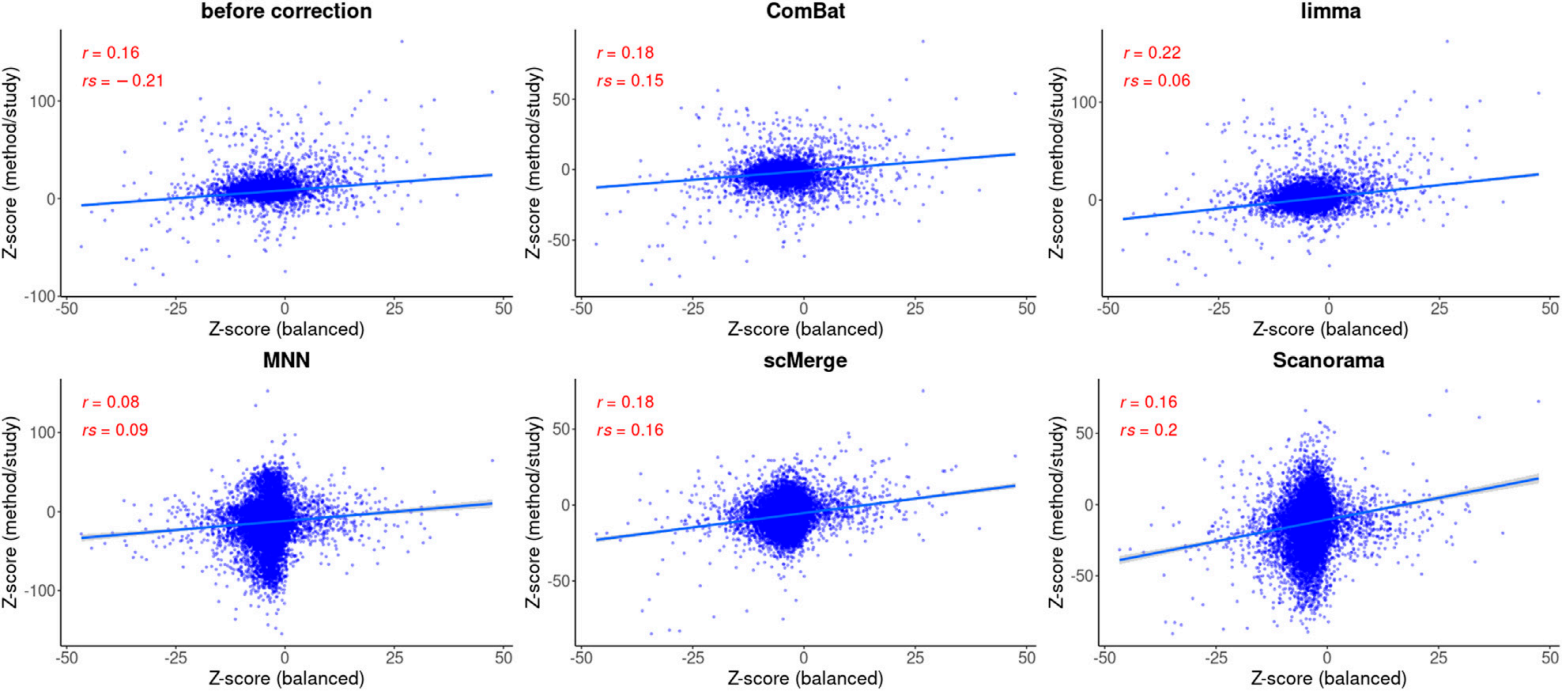
Correlation analysis after data integration using MAST statistics. Two correlation coefficients are shown: Pearson (R) and Spearman (*ρ*) and the corresponding *p* values. The regression model is fitted (blue line) with confidence intervals (the grey area around the line).

**FIGURE 6:**
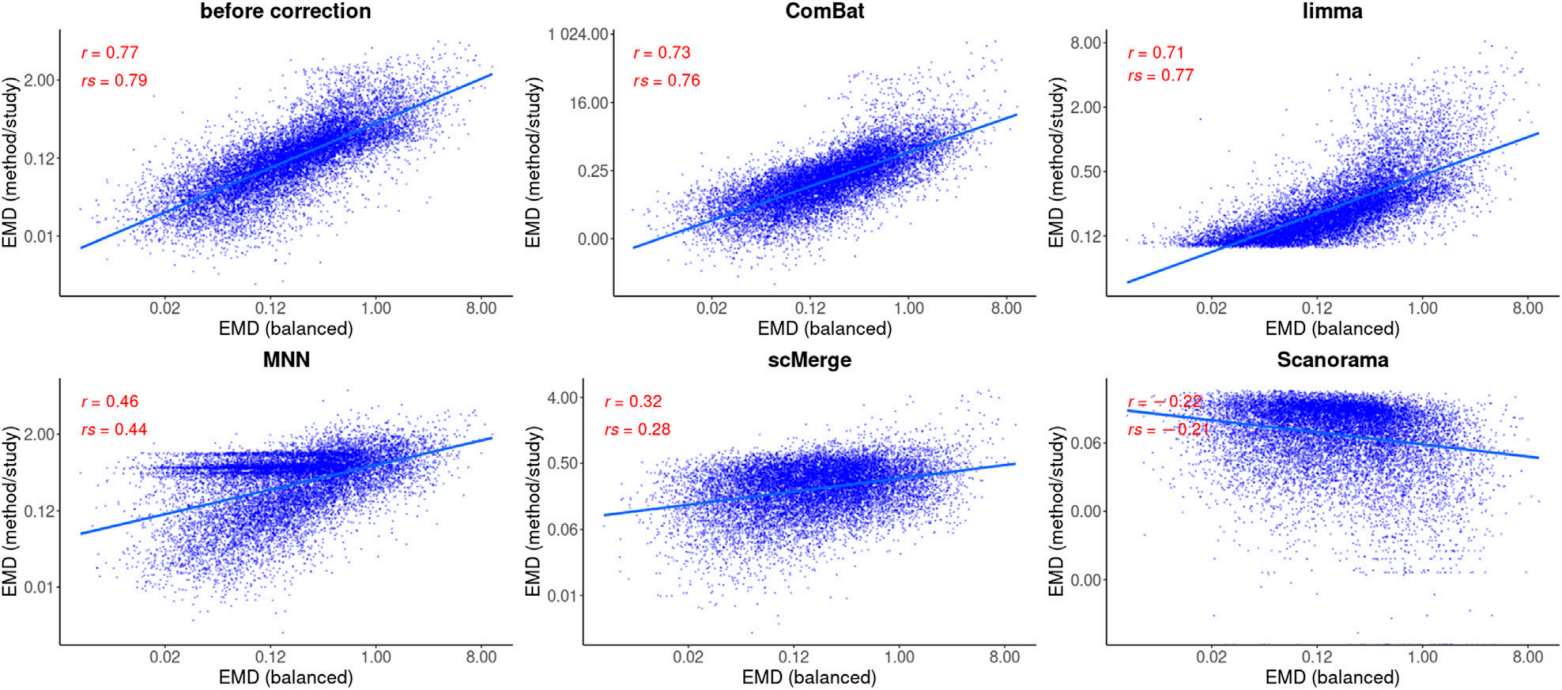
Correlation analysis after data integration using EMDomics statistics. Two correlation coefficients are shown: Pearson (R) and Spearman (*ρ*) and the corresponding *p* values. Regression model is fitted (blue line) with confidence intervals (grey area around the line).

For each method, only the best DEGs finding results were shown from all the setups tested (Figures 5, 6): MNN and Scanorama were run with all genes as input and scMerge with K4444 setting. The number of DEGs identified with MAST (parametric approach) and EMDomics (non-parametric approach) is presented in Table 2. The intersection between different data integration methods and approaches for DEGs finding was small. For the confounded study, the number of identified DEGs was almost identical between the two approaches, but the common part consists of only 62 genes (Table 2). After data integration, only ComBat-seq gave a higher number of DEGs than other methods, mostly when the parametric approach was used. The non-parametric approach identified a significantly larger number of DEGs after correction for other data integration methods.

**TABLE 2:** Number of DEGs after correction.

Study/tool	[MAST]	[EMDomics]	Intersection
balanced	965	328	246
confounded	191	197	62
ComBat-seq	287	115	114
limma	9	206	9
MNN	6	137	6
scMerge	0	20	0
Seurat	0	0	0
Scanorama	0	0	0

Based on the Pearson correlation coefficient (R), there is an improvement in the correlation of MAST DE statistics between the reference and the corrected study in the case of ComBat-seq, limma, and scMerge (Figure 5). For MNN and Scanorama, the test statistics themselves were much higher, thus the correlation with the reference is smaller (Figure 5). When the Spearman correlation coefficient is considered (*ρ*), the correlation is higher for every integration method, and Scanorama, scMerge, and ComBat-seq are the best. For a non-parametric test approach, after data integration, both correlation coefficients were smaller in all cases (Figure 6). However, ComBat-seq and limma showed the smallest decrease, while Scanorama gave negative correlation values. In some cases, rank-based EMDomics gave the same value of test statistic (dots arranged in horizontal lines in Figure 6), which follows from assigning the same expression values for individual genes after batch correction using selected methods (e.g., limma, MNN).

**FIGURE 7:**
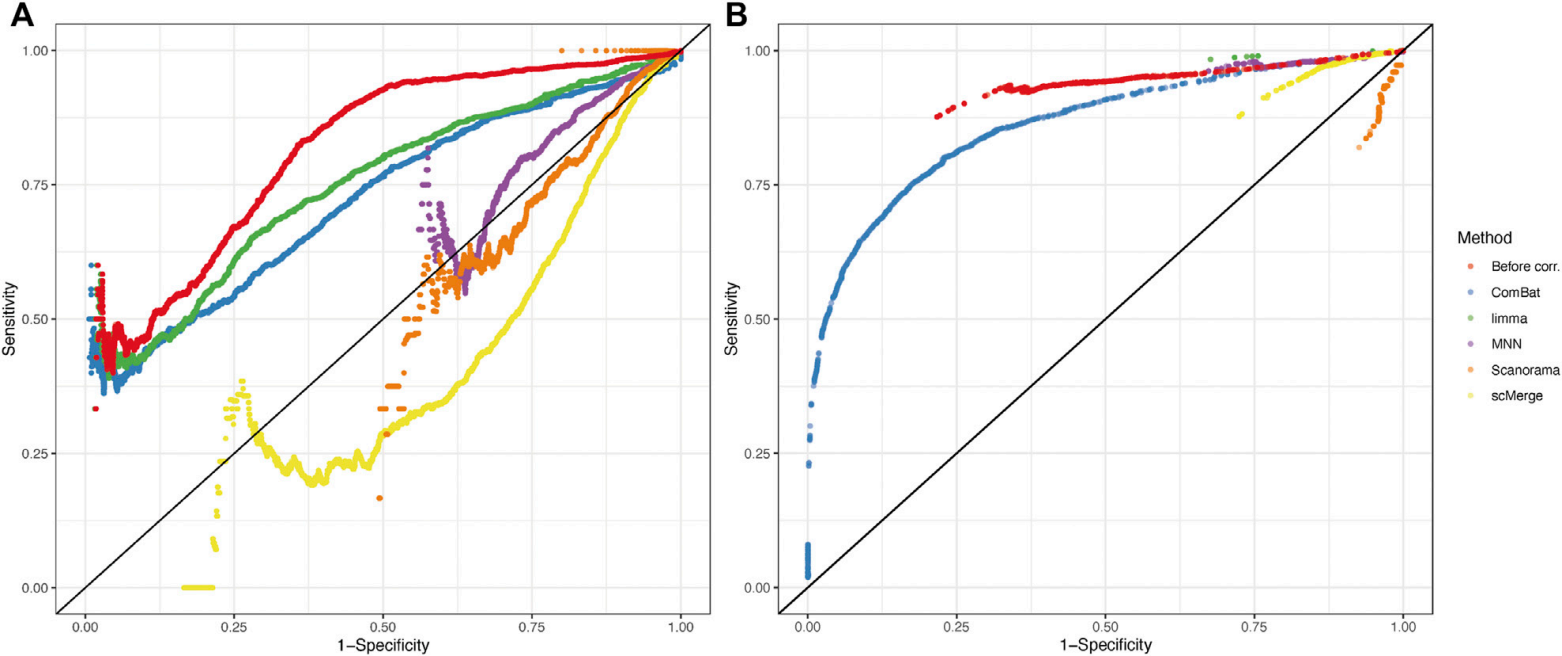
Receiver operating characteristic (ROC) curves from results of DEGs analysis using MAST **(A)** and EMDomics **(B)** tests. Color coding represents different data integration methods or no correction (red).

ROC curves calculated for each method and statistical tool (Figure 7) support the findings of correlation analysis. Only for ComBat-seq and limma, the area under the ROC curve was higher than 0.5 (Combat-seq: 0.72 and 0.86; limma: 0.74 and 0.65). The worst method was Scanorama (0.39 and 0.44).

### 3.5 Gene set analysis after data integration

**FIGURE 8:**
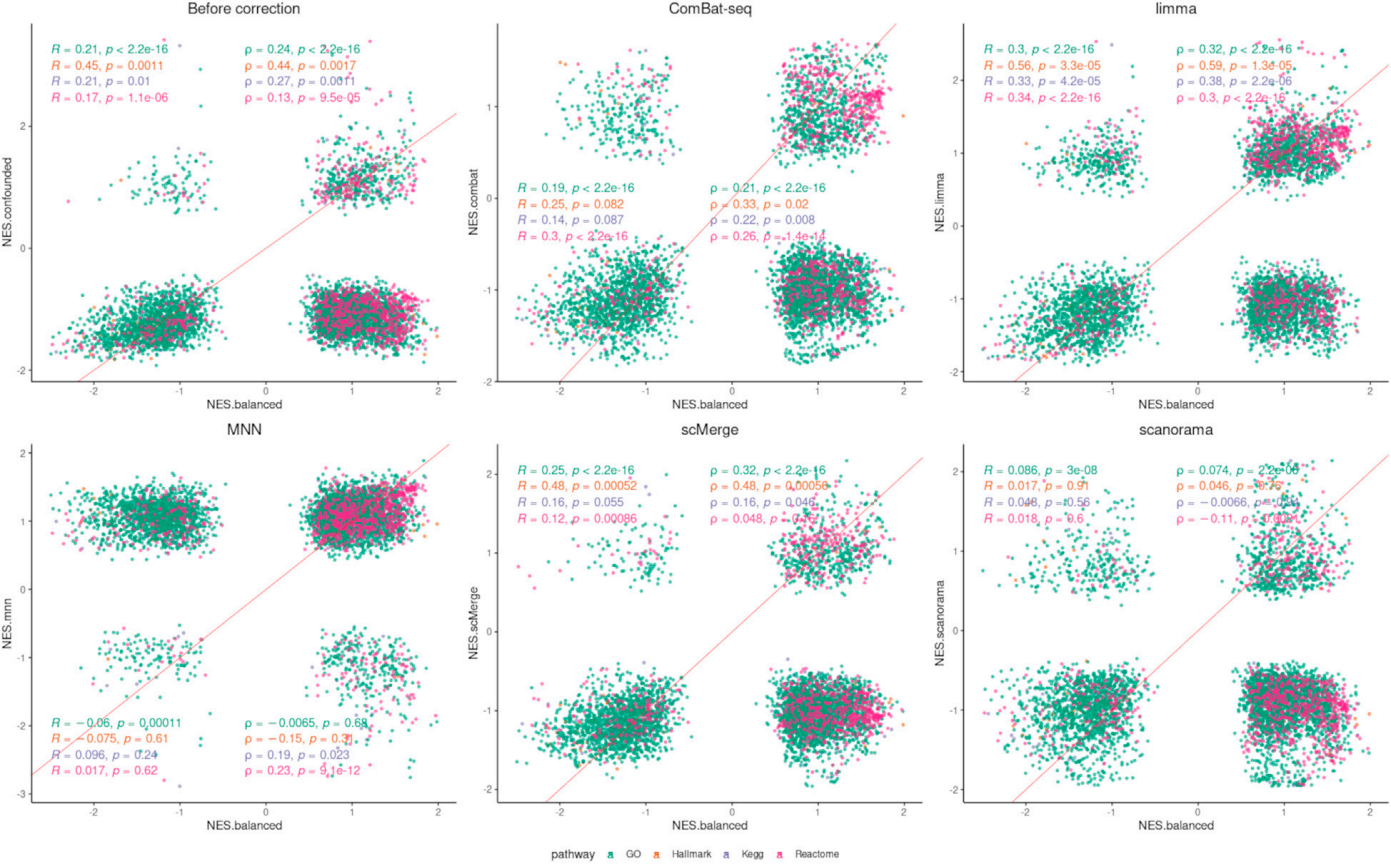
Correlation analysis (GSEA) after data integration using NES. Two correlation coefficients are shown: Pearson (R) and Spearman (ρ) and the corresponding *p* values separately for each pathway/gene set.

The number of significantly enriched pathways for selected gene sets is presented in Table 3. Overall, a smaller number of enriched pathways was found after correction. Data integration using ComBat-seq did not improve the correlation coefficients for any of the considered gene sets (Figure 8; Supplementary Table S1), but the dissimilarity was small. The opposite is observed in the case of limma, where the correlation improvement was found for all gene sets and both coefficients. scMerge improved both coefficients for Hallmark and GO and worsened for KEGG and Reactome. MNN and Scanorama worsened the correlation for every gene set.

**TABLE 3:** Number of enriched pathways for selected gene sets.

Study/tool	Hallmark	KEGG	GO	Reactome
balanced	31	19	568	219
confounded	21	14	387	51
ComBat-seq	0	0	25	13
limma	12	7	153	29
MNN	3	8	77	51
scMerge	4	3	46	16
Seurat [CCA]	15	9	326	10
Seurat [rPCA]	16	3	235	71
Scanorama	0	5	59	5

## 4 Discussion

We tested six scRNAseq data integration methods against two experimentally derived datasets which, in some sense, are mirror images of each other. Both experiments had the same biological properties such as cell line, drug, time of harvesting, etc. The only difference was in the technical study design; one experiment was designed to minimize the technical variation and was our reference, while the other manifested strong batch effects due to the difference in capturing time of each batch. This dataset was corrected for batch removal. Our study was not intended to evaluate many aspects of the batch correction (accuracy, speed, scalability) as other published benchmarks, but is focused on one unexplored so far aspect of scRNAseq data integration which is its impact on DGE analysis in real data scenario. Available benchmarks also address this problem, however, based only on the simulated data scenarios. While these evaluations can easily compute the number of true/false positive DEGs identified in corrected datasets, they do not stress the real challenge behind DGE analysis on batch-corrected datasets by excluding multiple technical and biological factors occurring in real data. For example, R package splatter (Zappia et al., 2017) simulates the batch effect by randomly generating multiplication factors from a log-normal distribution for each gene and group of cells (i.e., batch). However, since all cells within a batch are modified in the same way, parametric statistical tests can easily handle these artificial batch effects by adding covariates to the model. Thus, our study is unique and extends previous comparisons.

In this work, we tried to emphasize the challenge involved in feature-level analyses on corrected gene expression matrices. Indeed, cell-level analyses which are based on computing the distance (clustering or trajectory analysis) are safe to apply to corrected data because all cells are placed in the same coordinate space, which is the idea of data integration. However, integration algorithms give no guarantee to preserve relative differences in gene expression space. Therefore, correction methods may introduce artificial differential expression between cell types or conditions. Moreover, a majority of integration tools change the original nature of scRNAseq data: counts are no longer counts. One exception is ComBat-seq which preserves the integer nature of counts. Counts preservation is important for the compatibility of a corrected matrix with the available tools for differential expression analysis which may require counts or values equivalent to counts. A natural consequence of subtracting expression during integration (for example in MNN or Scanorama) is negative values in the corrected matrix which are hard to biological interpretation. Moreover, the scale of corrected values can be much different from the original counts which were especially apparent for Scanorama. Therefore, corrected values can no longer be considered as expression measures (of course still higher values reflect higher expression). Model-based methods specifically designed for scRNAseq DGE analysis (parametric approaches) may not work well with corrected data given the fact that many properties of original data are lost, and higher expressed genes are dragged down after correction. Of course, one can attempt to apply some transformations (e.g., Box-Cox transformation) on corrected data, but they are computationally intensive and do not guarantee the intended effect.

In general, gene set enrichment analysis should be more robust against batch correction than gene level analysis but in our case, this was not manifested. ComBat-seq which was best on DGE analysis (in both, number of DEGs and correlation with balanced study) did not improve correlations on the level of gene sets, but it also did not decrease it much.

In terms of computational time, limma was the fastest algorithm, while Scanorama used the least amount of memory (Table 4). MNN ran on 8 processor cores, was much slower than others (even algorithms ran on a single core) and in peaks, it needed almost 30 GB of memory. We summarized all our findings when comparing data integration methods in Table 4. Our evaluations were done on a machine with Intel^®^ Xeon(R) CPU E5-2,650 v3 at 2.30GHz × 40 and 256 GB RAM.

**TABLE 4:** Summary of comparison between data integration methods.

Method	Total time [sec]	Single core time [sec]	Total RAM [MiB]	Peak RAM [MiB]	Ease of use	Original data distribution	UMAP separation
ComBat-seq	4,007.2	4,007.2	2,039.5	30,652.1	easy	not changed	good
limma	24.2	24.2	2,039.2	15,302.1	easy	changed	medium
MNN (8 cores)	52,300.6	418,405	2,040.1	28,121.4	easy	changed	medium
scMerge (8 cores)	14,404.9	115,240	2,042.8	28,117.2	easy	changed	medium
Seurat (5 workers)	1,116.3	5,581.5	4,486.6	17,066.4	easy	changed	weak
Scanorama	5,925.9	2,542.7	2,055.4	2,055.6	medium	changed	weak

Our study has some limitations. First, the analysis was done on a set of two experiments concerning the same cancer cell line. The results might slightly differ for other organisms. However, since there is no other pair of experimentally derived balanced/confounded studies, it was not possible to test it. Second, different methods have multiple parameters to set. We have chosen default values where possible and tested a few settings for another method, however, we are aware that the optimal settings might not be reached in this study.

Finally, we are rather careful with formulating overall recommendations for the particular method as well as we do not state that DGE analysis should not be performed at all. We rather wanted to highlight the fact that single-cell data integration is one of the current grand challenges (Lahnemann et al., 2020) in omics analyses and better methods might still appear. Nevertheless, we wanted to highlight the ComBat-seq method as it led to the highest correlation of test statistics between reference and corrected dataset among others and it does not distort the original distribution of gene expression, so it can be used in all types of downstream analyses.

## Data Availability

Publicly available datasets were analyzed in this study. This data can be found here: [Reference study]: https://doi.org/10.3390/cancers12092551 [Test study]: https://doi.org/10.1038/s41523-021-00270-4.
