# Supplementary material for: Influence of single-cell RNA sequencing data integration on the performance of differential gene expression analysis
Source: Front Genet. 2022 Nov 1;13:1009316. doi: 10.3389/fgene.2022.1009316 (PMC9663917; doi:10.3389/fgene.2022.1009316)
Supplement: Supplementary file 1 [file Table1.docx]

Supplementary Table 1. The number of genes in each quadrant of Figure 8, after GSEA.

| **NES sign in balanced/confounded study** | **Before correction** | **ComBat** | **limma** | **MNN** | **scMerge** | **Scanorama** |
| --- | --- | --- | --- | --- | --- | --- |
| **-/-** | 1634 | 1460 | 1389 | 142 | 1617 | 1455 |
| **-/+** | 103 | 277 | 348 | 1595 | 120 | 282 |
| **+/-** | 2859 | 2305 | 1875 | 370 | 2740 | 2680 |
| **+/+** | 542 | 1096 | 1526 | 3029 | 661 | 721 |
