# Supplementary material for: Influence of single-cell RNA sequencing data integration on the performance of differential gene expression analysis
Source: Front Genet. 2022 Nov 1;13:1009316. doi: 10.3389/fgene.2022.1009316 (PMC9663917; doi:10.3389/fgene.2022.1009316)
Supplement: Supplementary file 2 [file Image1.pdf]

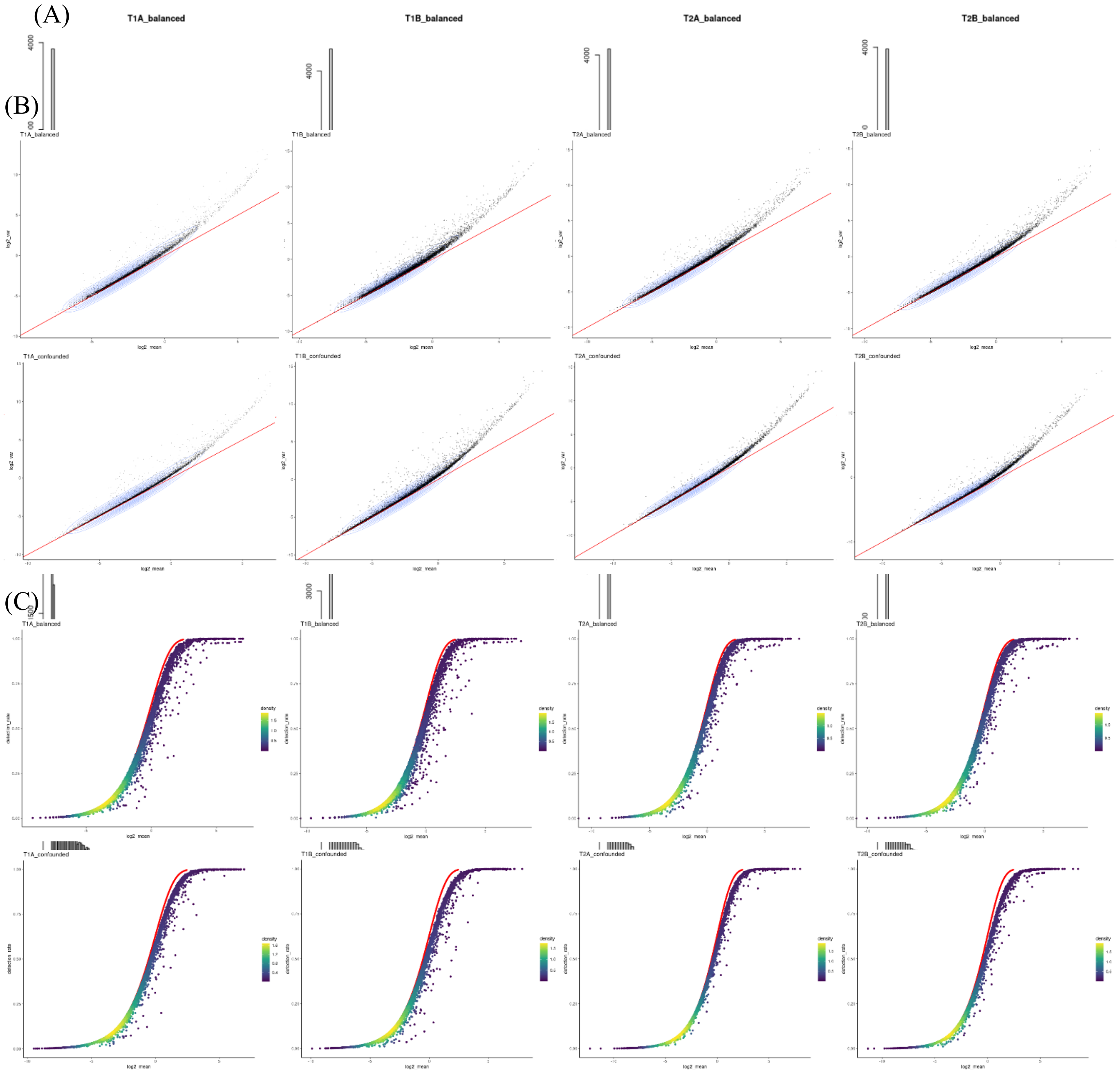

Supplementary Figure 1. Feature-level metrics for balanced and confounded (before correction) study. (A) histograms of mean expression, (B) mean-variance relationship, (C) mean-detection rate relationship
